# Learnings from the design and acceptance of the German COVID-19 tracing app for IS-driven crisis management: a design science research

**DOI:** 10.1186/s12911-021-01579-7

**Published:** 2021-08-09

**Authors:** Alina Behne, Nicolai Krüger, Jan Heinrich Beinke, Frank Teuteberg

**Affiliations:** 1 Institute Accounting and Information Systems, Osnabrück University, Katharinenstr. 1, 49074 Osnabrück, Germany; 2 Smart Enterprise Engineering, German Research Center for Artificial Intelligence, Parkstraße 40/42, 49080 Osnabrück, Germany

**Keywords:** Crisis management, Corona-Warn-App, Tracing apps, Design science, User experience design, Prototype

## Abstract

**Background:**

This article investigates the research problem of digital solutions to overcome the pandemic, more closely examining the limited effectiveness and scope of the governmental COVID-19 tracing apps, using the German COVID-19 tracing app (Corona-Warn-App) as an example. A well-designed and effective instrument in the technological toolbox is of utmost importance to overcome the pandemic.

**Method:**

A multi-methodological design science research approach was applied. In three development and evaluation cycles, we presented, prototyped, and tested user-centered ideas of functional and design improvement. The applied procedure contains (1) a survey featuring 1993 participants from Germany for evaluating the current app, (2) a gathering of recommendations from epidemiologists and from a focus group discussion with IT and health experts identifying relevant functional requirements, and (3) an online survey combined with testing our prototype with 53 participants to evaluate the enhanced tracing app.

**Results:**

This contribution presents 14 identified issues of the German COVID-19 tracing app, six meta-requirements, and three design principles for COVID-19 tracing apps and future pandemic apps (e.g., more user involvement and transparency). Using an interactive prototype, this study presents an extended pandemic app, containing 13 potential front-end (i.e., information on the regional infection situation, education and health literacy, crowd and event notification) and six potential back-end functional requirements (i.e., ongoing modification of risk score calculation, indoor versus outdoor). In addition, a user story approach for the COVID-19 tracing app was derived from the findings, supporting a holistic development approach.

**Conclusion:**

Throughout this study, practical relevant findings can be directly transferred to the German and other international COVID-19 tracing applications. Moreover, we apply our findings to crisis management theory—particularly pandemic-related apps—and derive interdisciplinary learnings. It might be recommendable for the involved decision-makers and stakeholders to forego classic application management and switch to using an agile setup, which allows for a more flexible reaction to upcoming changes. It is even more important for governments to have a well-established, flexible, design-oriented process for creating and adapting technology to handle a crisis, as this pandemic will not be the last one.

**Supplementary Information:**

The online version contains supplementary material available at 10.1186/s12911-021-01579-7.

## Background

The world has been facing the COVID-19 pandemic since 2019 (amounting to 140,322,903 confirmed COVID-19 infection cases^1^ worldwide). While governments are striving to achieve the most promising strategies to lower infection rates, such as via lockdowns, the full potential of digital tracing apps has not yet been realized in many countries. One existing solution for contact tracing lies in the Exposure Notification Framework (ENF) published by Apple and Google.^2^ In Germany, by its federal state structure, all corona-related non-pharmaceutical interventions, such as local curfews or rules for schools and kindergartens, are decided by each federal state [2]. Thus, the Corona-Warn-App (henceforth the CWA), which is built on the ENF, is the only nationwide response to the current situation in Germany. Therefore, the CWA faced the highest expectations from the beginning and raised several ethical or privacy-related concerns prior to its launch [40]. With a target group of more than 80 million users, the technical aspects, such as the requirements for accessibility, connectivity, and user experience, were quite ambitious. Thus far, the recommended mark of 60% of users (to achieve a significant benefit regarding the infection tracing) among the population has not yet been reached, with the current 23.8 million users [19, 46].^3^ Furthermore, epidemiologists recommend lowering the incidence value (i.e., new coronavirus disease 2019 infections in a given timeframe for a population) [45, p. 77], to an incidence value of around 25 to disburden local health departments in contact tracing, which could serve as the primary benefit of a digital tracing solution [26]. Thus, there remain open research fields in terms of mass acceptance and the design criteria of the CWA. From a practical viewpoint, the Robert Koch Institut (RKI) published a job vacancy for the CWA advancement during the research timeframe of this paper, indicating needed support in this area.^4^ Complementary research from different disciplines and perspectives, such as epidemiology, mathematical modeling, or information systems, is required to help fight the pandemic [53] and is highly welcome, as proclaimed at the top of the official CWA website (see Fig. 7, “Appendix”).^5^

As COVID-19 tracing is a highly sensitive topic, a large bandwidth of publications concerns possible failures in terms of privacy, security, data accuracy, or epidemiological effects (e.g., [20, 21]). Nevertheless, we identified a research gap in a design-oriented, forward-driven approach in developing, designing, and testing concrete enhancements of existing COVID-19 tracing apps (here, the German Corona-Warn-App). Various authors (e.g., [4, 54]) examine prototypes and vignettes regarding their acceptance, but field studies based on the released application have not yet been published. Therefore, to enhance the design and features of this app and its acceptance, this paper addresses the research gap after the launch and diffusion of the German COVID-19 tracing app.

The potential for improvement of the CWA is evident by comparing the shared tests (56,681) and not-shared positive tests (43,631) for the CWA with the corona-positive tested persons (583,622) in Germany [8, 47]. After all, the number of shared test results outweighs the number of non-shared tests, but the difference in COVID-19-positive non-users of approximately 500,000 people is significant. Thus, this paper aims to address this problem by increasing the acceptance and knowledge regarding the functions and meaningfulness of the app based on a prototypical agile enhancement. In addition to our online survey consisting of 2616 participants from Germany in July 2020, generating plenty of feedback and ideas for further development, we have used renowned practical literature and podcasts from well-known epidemiologists (such as Prof. Dr. Drosten and Prof. Dr. Lauterbach) and the advice of primary experts from the healthcare and IT sectors. Therefore, we investigate the following research questions (RQs):

*RQ1:* Which design and functional elements of the German COVID-19 tracing app (Corona-Warn-App) should be enhanced to increase its acceptance?

*RQ2:* What lessons can be learned for applications in crisis management from the challenges of the Corona-Warn-App?

The remainder of this paper is structured as follows: First, we describe the research field and discuss related work. Following this, “Multi-methodological approach” section contains the method of this paper: a design science research approach. In “Results: artefact description” section, we present our prototype. Furthermore, the evaluation of the current CWA and our extended version within three cycles are described in “Evaluation as Part of the Development” section. Based on our results, “Discussion and implications” section provides the practical and scientific implications and a new user journey regarding an enhanced CWA. In addition, we derive general learnings for applications in crisis management. Finally, in “Conclusion and outlook” section, we summarize our results and implications, drawing a conclusion.

Research concerning COVID-19 tracing apps is rapidly expanding. In the field of information systems (IS), multiple research perspectives have evolved—for example, technical specifications such as infrastructure [1, 24, 55], acceptance (e.g., [4, 10, 38, 39, 54], and data security (e.g., [6, 11, 22, 50, 52]). Due to the topicality and the publication pipelines, the research data of these papers is primarily built upon vignettes or prototypes of hypothetical COVID-19 tracing apps (e.g., [4]). Jahnel et al. [23] discuss the data privacy dilemma of corona-tracing apps, particularly for the German COVID-19 tracing app, and conclude that the impact and effectiveness of the CWA for the pandemic situation cannot be measured. High privacy standards are needed to achieve a certain level of acceptance in the public, however, in retrospect, this high standard limits the evaluability of this technology. For future app development in this context, the authors suggest stronger user involvement and voluntary donation of data [23]. A clear focus on the deployed CWA acceptance, front-end, and use itself has been published by Lasarov [32]. In his research, Lasarov conducted a sentiment analysis of approximately 1000 comments regarding the CWA and derived three leveraging categories for the acceptance of the app: (1) usage benefit, (2) data protection (and the related factors privacy, transparency, and trust) and (3) social implications.

In global crisis management, the potential enhancement of the CWA with design science is a valuable investigation that should be shared with the community [53]. Many previous research articles primarily focus on privacy aspects, for example, Stroscher et al. [52] conduct a privacy assessment on the CWA, which could be treated as one aspect of CWA design. In contrast with other research methods, both the scientific outcome and the communication to the public are components of the entire research journey. As the need for broader scientific communication increased also in the IS discipline, design science research (DSR) offers, on one hand, a rigorous and well-elaborated process for IS research. On the other hand, scientific implications do not stand alone as the generated knowledge to be appended to the body of knowledge, but—according to Hevner et al. [18]—creating a communication concept is part of the scientific responsibility.

**Fig. 1:**
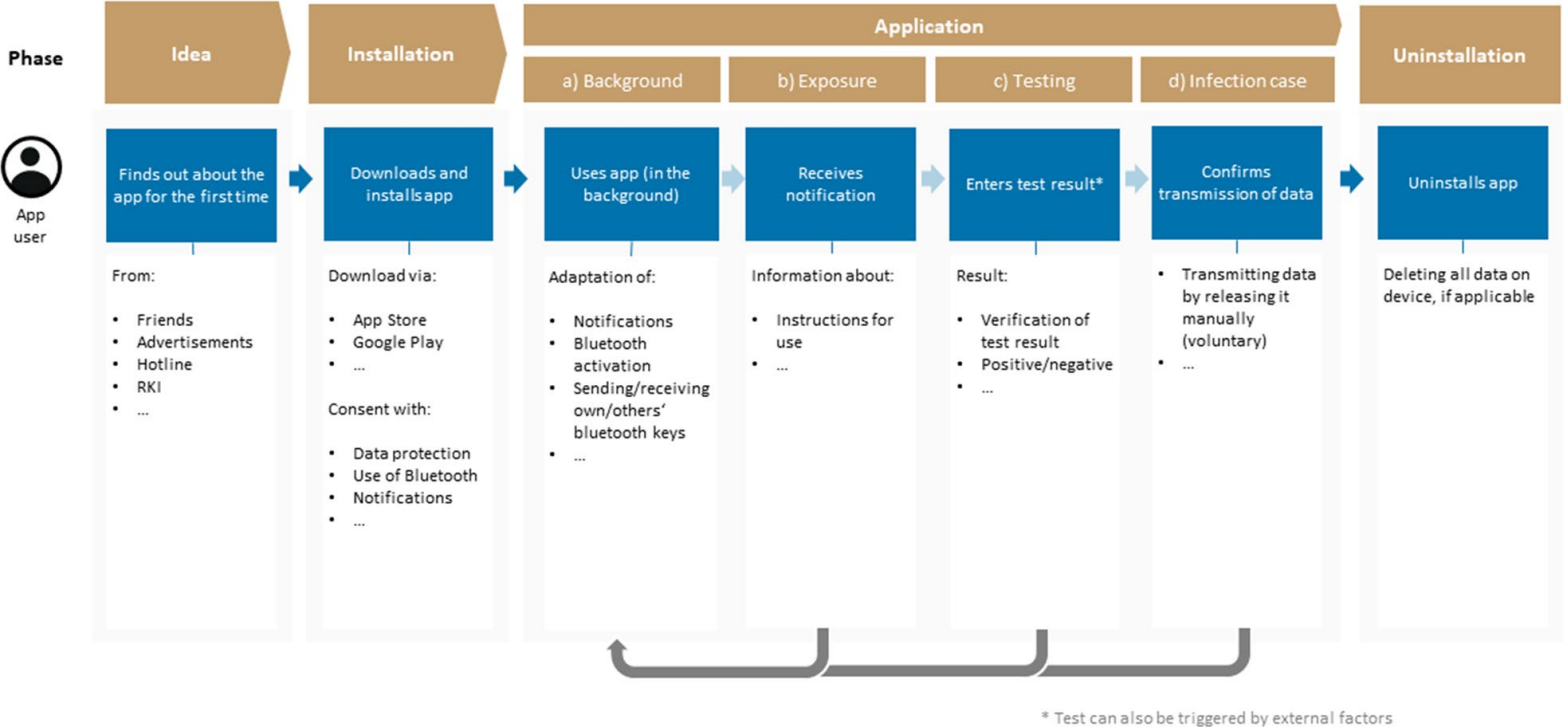
User journey of the first version of the Corona-Warn-App^5^

To strengthen the argument that a DSR approach is recommendable and reasonable in this context, Fig. 1 presents the original user journey^6^ of the CWA, published by the SAP^7^ developer team on Github. It is noteworthy that the CWA was the first worldwide open-source published implementation of the Google/Apple framework, and therefore, it can be understood as an important reference (and blueprint) for other countries. Thus, the limited scope of the user journey must be reflected critically within this paper, as it was not updated by the developers since the first design draft. We question whether this approach is still recommendable in the third wave of the pandemic. Regarding the rush within the development process, we build our research design on potential enhancements: First, as outlined in the user journey, the CWA has been designed in a linear, not a circular, flow. This can be described along a phenomenon in software development, Conway’s law [7]: Organizations design systems with the tendency to represent existing structures rather than follow the agile paradigm to design a user-centered application and adapt internal structures and data flows to these needs. Second, the current user journey simply neglects several use case scenarios, which are part of the medical path that users of the app experience: People overcome a coronavirus disease 2019 infection, but this scenario is not illustrated. Even the official CWA website admits that in the case of recovery, the user must reconfigure the app manually.^8^

## Multi-methodological approach

**Fig. 2:**
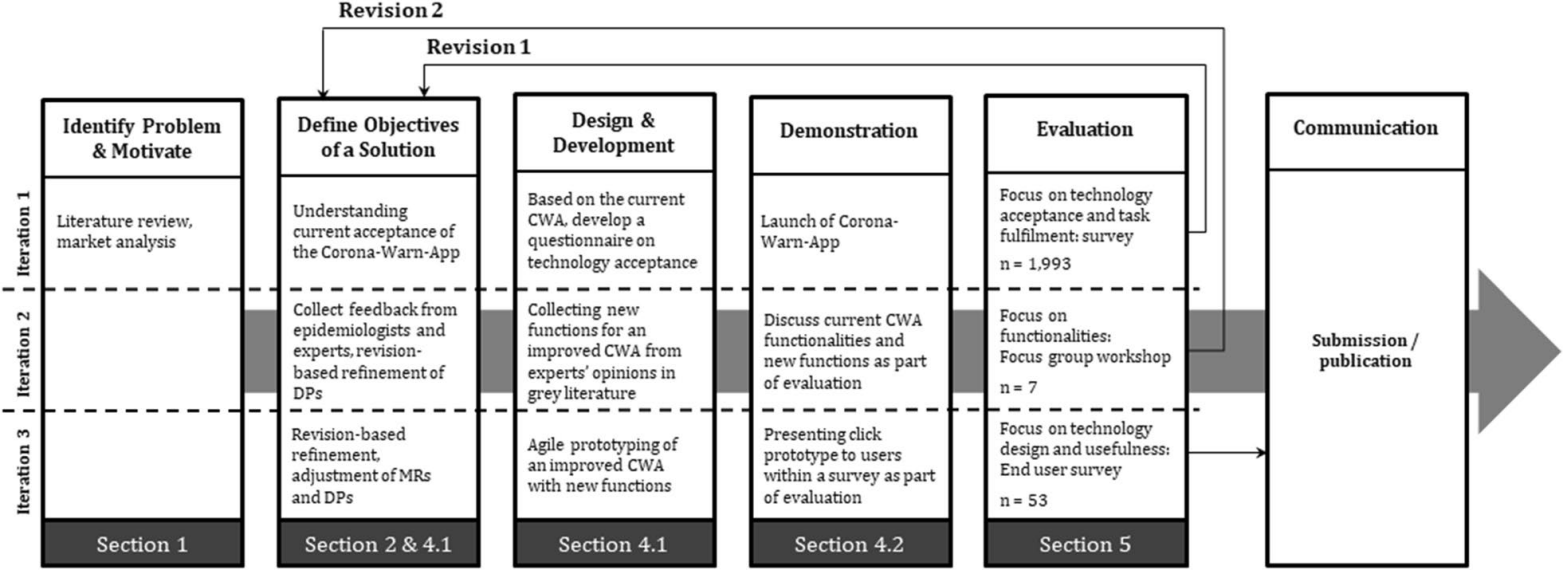
Design science-based research approach

As presented in “Background” section, several investigations concerning tracing apps already exist. Nevertheless, there is a research gap for DSR applied to crisis management. This motivates us to investigate feedback regarding the CWA from users, epidemiologists, other health experts, and IT experts. This DSR project is part of a 7-month investigation examining the technology acceptance of the CWA and furthering the development of a prototype with additional functionalities, for which it was valuable to build upon the results of the acceptance study in German society. We followed the DSR paradigm of Hevner et al. [18] and derived meta requirements and design principles. Furthermore, we enriched this approach by preparing and presenting the results in agile development terms for further procedures. Thus, in addition to deriving meta requirements, we included more detailed, functional requirements so that these results could be directly applied in practice. In doing so, we added the DSR view of Peffers et al. [41] to our research: Communicating research artifacts—to the public and professionals—has great importance in the Peffers et al. [41] DSR approach. In the given context, the contribution of this research should be communicated toward app- and policymakers and the open-source community on Github. Figure 2 presents three design cycles and respective evaluation cycles to ensure the identification of any potential for improvement, especially for the enhancement of the functionalities and design of the CWA. The applied procedure contains (1) a survey featuring 1992 participants from Germany for evaluating the current CWA, (2) a gathering of recommendations from epidemiologists in practical literature and from a focus group discussion with IT and health experts, prioritizing and identifying sophisticated functional requirements, and (3) a survey for evaluating the presented enhanced CWA. The survey structures and the interview guideline can be seen externaly (see Additional file 1).

Regarding the first iteration cycle, our initial starting point was the current version 1.0 of the CWA (until 12 August). We surveyed 2616 participants from Germany, in which we examined user acceptance, focusing on reasons for usage and non-usage. The data collection ended in the middle of July 2020; thus, the feedback pertains to the first month of using the CWA, since the app was published in June for the first time. We cleaned the data results by deleting incomplete questionnaire answers and those in which the time needed for the participant to complete the questionnaire was less than four minutes, yielding 1,992 relevant respondents of our survey. On one hand, we analyzed the data of our technology acceptance model (TAM) according to Davis [9] using partial least squares structural equation modeling (PLS-SEM) according to Hair et al. [17] using the analysis tool WarpPLS [27]. On the other hand, we investigated the descriptive results (see Table 4, “Appendix”). As a result, we received feedback and recommendations for improvement from a user’s perspective (see Table 3).

In the second iteration cycle, a set of features was derived to enhance the effectiveness of the CWA from an epidemiological perspective: We extended the feedback of the first round with expert opinions, such as epidemiologists from podcasts (NDR Corona-Virus-Update, UKW, Kekulés Corona Kompass) and online news (e.g., [33]). Therefore, to obtain a comprehensive understanding of possible features, an open Google search was performed using the following search string: ‘“Corona-Warn-App” AND update OR feature* OR “Design Science Research” OR DSR’. Subsequently, prior to performing the in-depth analysis, we coded each feedback into a matrix and assigned each feedback to a newly defined abstracted area (a functional requirement) and a category (an epic). This coding was conducted by two authors independently of each other with an inter-coder agreement of 0.81, which is greater than the defined threshold of 0.8 [30]. Different results were discussed and resolved by the authors. In total, summarizing our feedback into functional requirements for an enhanced CWA served as our basis for development (see “Prototype development: an enhanced COVID-19 tracing app” section  and Table 2). For further evaluation, we discussed and ranked CWA functions and potential future use with a focus group workshop according to Gläser and Laudel [14] (see “Evaluation cycle 2: usefulness and usability” section). Table 1 displays the seven participants involved in this focus group. Based on the ranked functional requirements of the second evaluation cycle, we developed our COVID-19 tracing app prototype using Adobe XD (see “Prototype development: an enhanced COVID-19 tracing app” section). This prototype contains new content management, crowd notification, mask obligation, vaccination, and corona test results that are not yet included in the current CWA. In the final evaluation, we conducted an online experiment with 53 end users. The questions were focused on the navigation of the app regarding the user experience according to Schrepp et al. [49]. Here, the participants were asked to solve five different tasks in the prototype (see “Evaluation cycle III: usability” section).

**Table 1:** Participants involved in the focus group

#	Description	Professional experience
E1	Digital lab member in a welfare association in healthcare	> 15 years
E2	Manager of a health app in the cardiovascular context	> 15 years
E3	Software engineer	> 1 year
E4	Physician and medical crisis coordinator	> 30 years
E5	Health department supporter of the city council	> 20 years
E6	Press officer of a welfare association in healthcare	> 20 years
E7	Business information systems engineer	> 15 years

For both surveys and all items, we used a seven-point Likert-type with multiple-item scales [36] and conducted pretests before the public survey began. As a result, we corrected linguistic mistakes and added more open questions in the event that the participants wanted to share their opinions. Regarding the data collection for all three evaluation cycles, we acquired people through social networks, personal email, and the newspaper.

## Results: artefact description

### From issues to design principles for the COVID-19 tracing app

**Fig. 3:**
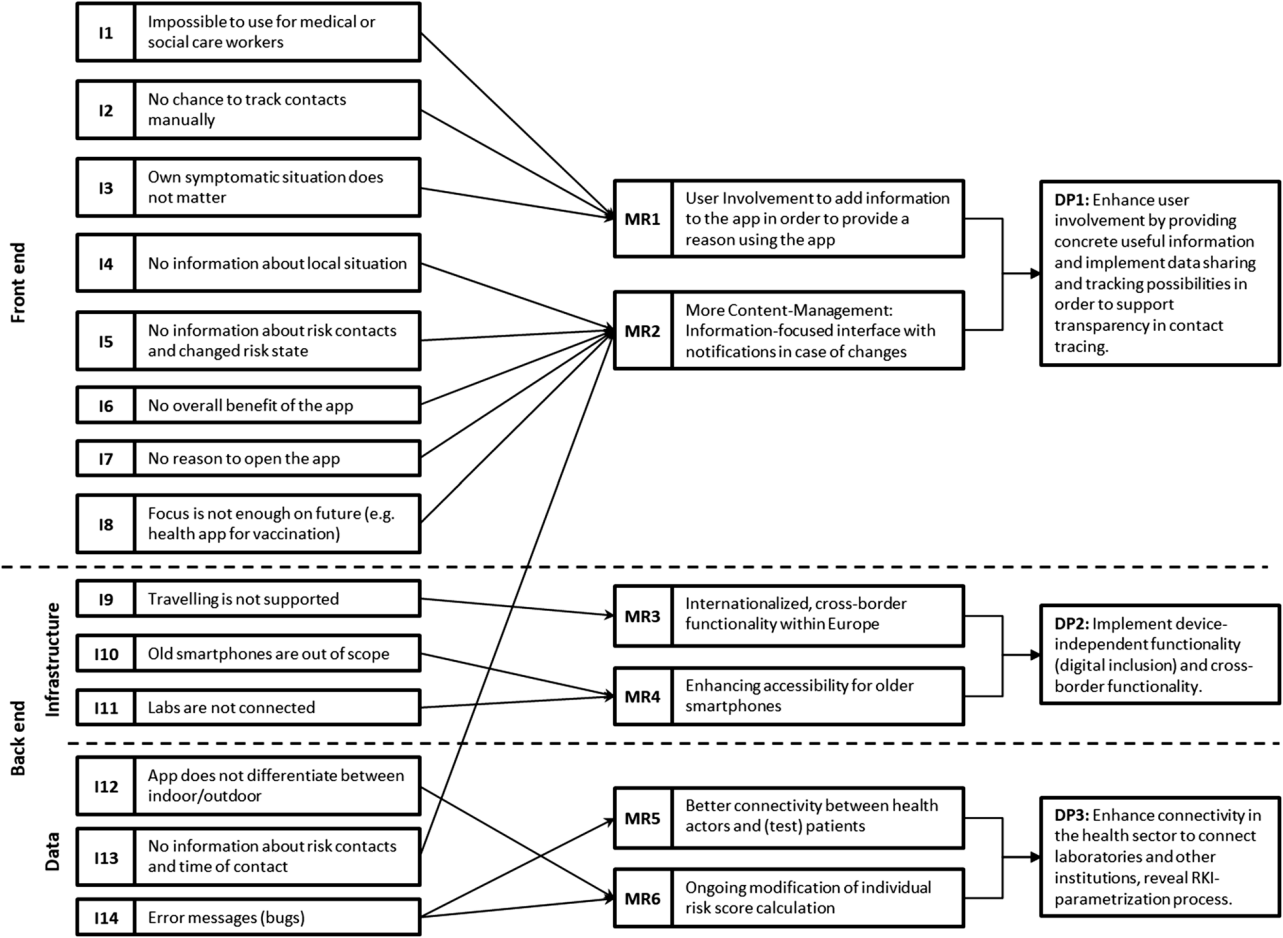
Derived issues, meta-requirements, and design principles

The greatest motivation to extend the CWA emerged from our survey investigating the acceptance of coronavirus tracing solutions. Thereby, we received ideas for several possible improvements and extensions for this app. Furthermore, as mentioned above, the app creators actively ask the expert and open-source community for assistance, where we can see a good match for the call for research in this journal from the scientific angle with the unique real-life situation under COVID-19. In addition, from the literature and our acceptance survey, we identified several issues of the CWA. In total, we counted 14 summarized issues, which result in functional requirements (I1–I14 cf. Figure 3, for references, see Table 2). From this perspective, we managed to derive six meta-requirements (MRs) and three design principles (DPs) (see Fig. 3), which were defined and evaluated by the last two iteration cycles and grouped into the front-end and back-end (data and infrastructure).

**Table 2:** Synopsis of derived features, clustered in Scrum-like epics and user stories

Epic	Example user story (as a user)	Functional requirement		Source
*Front-end*				
Content management	I want to see information about the local spread of the virus inside the app		Information on the regional infection situation	[16, 28, 43, 44]; E5, our first survey results
Content management	I want to have some basic information about contact with an infected person, as far as location and time are concerned		Information about contact cased	[29], our first survey results
Content management	I want to be able to learn the current best practice behavior inside the app		Education and health literacy	Pritlove and Mayer ([43]; E1)
Cluster and event recognition	I want to document my participation at an event (with given privacy)		Crowd and event notification	[37, 42, 43, 48]
Cluster and event recognition	I want to know upfront when I enter a crowd and post when I have been in a potential cluster		Cluster recognition	[28, 29]
Cluster and event recognition	I want to be warned in situations with many people		Crowd sensitivity	Kriehn ([29]; E7)
COVID-19 test result report	After quarantine, I want to clean up my positive test result		Clean up test results after infection	Our first survey results
Diary	I want to document my symptoms inside the app and report them		Tracking of own symptoms	Köver and Beckedahl ([28]; E6)
Diary	I want to do a daily write-up of all situations of the day, in which I was close or even too close to other people		Contact diary	[13, 29]
Regular use	I want to configure what happens in an automated detected or manual parametrized at-work mode		At-work mode	Kriehn ([29]; E5)
User experience	I want to have a reason to use/open the app (e.g., daily)		Gamification	Pritlove and Mayer [43]
User experience	I want the app to better display what data is being used and how it is being collected. This would greatly increase awareness of when data is being sent		Privacy concerns	Our first survey results
Vaccination	I would like to get vaccinated and document the vaccination date and location		Vaccination	(E4)
*Back end*				
COVID-19 test result report	I want to receive my test result via the app, regardless of where I took the test		Connecting labs	Köver and Beckedahl [28]; our first survey results)
Regular use	I want the app to update more often		Update more regularly	Our first survey results
Parameterization	I want to understand the risk calculation of the app to estimate my risk status		Ongoing modification of risk score calculation	[58]
Parameterization	I want the app to distinguish between indoor and outdoor contact situations		Indoor versus outdoor	Pritlove and Mayer [43]
Accessibility	I want to use the app when abroad, and I want foreign visitors to use their local app with a connection to the same warning server		Cross-border functionality within Europe	Whitelaw et al. [57], E1, our first survey results
Accessibility	Users with an old/no smartphone: I also want to participate in the CWA approach		Compatibility with older smartphone/offering separate technologies	[3, 28], [12]; E4

On the front-end side, our review of practical articles and the survey revealed a significant blind spot of the app for healthcare workers (**I1**): While this cohort expresses the strong intention to use the app, their professional environment makes this impossible, as they are dealing with infected people all day long but under the strongest possible safety measures. In contrast, from the broader perspective of general users, several epidemiological experts argue toward the issue (**I2**) that manual contact documentation is not within the scope of the current app. This might overcome data privacy issues, as manual documentation in a diary style would not create any privacy threats. Furthermore, a daily documentation routine of the user might be linked with a symptom diary (**I3**), which is currently a major problem for health authorities, when possibly infected people must remember contacts and symptoms in a timeframe of around 14 days. Summarizing this in a meta-requirement [**MR1**], the overall necessity for more enhanced user involvement can be derived. In the simplest understanding of this MR, the user needs a reason to open the app [**I7**], which is currently not the case, as—under normal (i.e., error-free) conditions—the app runs in the background. The corona-data experts of the UKW podcast [43] even argue for a gamified UX. Regarding content-driven use cases of the CWA, the data sources from our initial survey and the literature indicate the need for further coronavirus information [**I4-6**]. Furthermore, future-oriented features, which face the fact that the pandemic will not end shortly and might not be the last one, should be included [**I8**]. The second meta-requirement addresses this [**MR2**]: enhanced and localized content management, containing an information-focused interface with notifications in case of changes. Based on the aforementioned issues (I1–I14) and MRs (MR1–MR6), the following three DPs should be considered: ***DP1*** Enhance user involvement by providing concrete useful information and implement data sharing and tracking possibilities to support transparency in contact tracing.

Considering the user needs from an infrastructure perspective, we derived three issues from the data and literature [**I9–11**] expressing the strong wish of users and experts to invest more into a European-wide coronavirus warning system based on the Google-/Apple Framework (which represents the core functionality of the CWA). This yields **MR3**: internationalized, cross-border functionality within Europe. Moreover, as a second inclusion factor, **MR4** can be connected to this: Enhancing accessibility for older smartphones. This should not only include the backward compatibility of the smartphone OS but also a fall-back safe infrastructure for coronavirus testing centers and labs, granting everyone a comfortable and safe way of receiving coronavirus testing results. Concluding these requirements in an abstracted design principle, we recommend ***DP2***: Implement device-independent functionality (digital inclusion) and cross-border functionality.

As a final layer, issues surrounding data handling can be clustered [I12–14], which was again derived from both practical expert articles and the survey itself. **MR5** builds upon the aforementioned MR4 but adds the problem of wider digitalization: Improved connectivity between health actors and (test) patients. The last meta-requirement **MR6** addresses a practical issue (especially **I12**). However, it must be viewed as a multidimensional problem of epidemiological and technical questions: Parameterized by the ENF by Apple and Google and localized for Germany by the RKI, this issue is multifarious.^9^ MR6 aims to achieve an ongoing modification of individual risk score calculation. Currently, this setting is indeed published and open-source; however, it is only read and understandable by a few in-depth experts. Finally, the following design principle can be derived: ***DP3*** enhance connectivity in the healthcare sector to connect laboratories and other institutions, revealing the RKI parametrization process.

### Prototype development: an enhanced COVID-19 tracing app

Initiated by the design principles, we designed an enhanced CWA prototype.^10^ This was developed using several functional requirements, as presented in Table 2, which were derived with the help of identified issues (I1–I14) out of the evaluation steps. Regarding agile development, functional requirements can be assigned to an epic—a category of functions regarding one theme. To take the functional requirements one step further into an agile development setting, we formulated a user story.

**Fig. 4:**
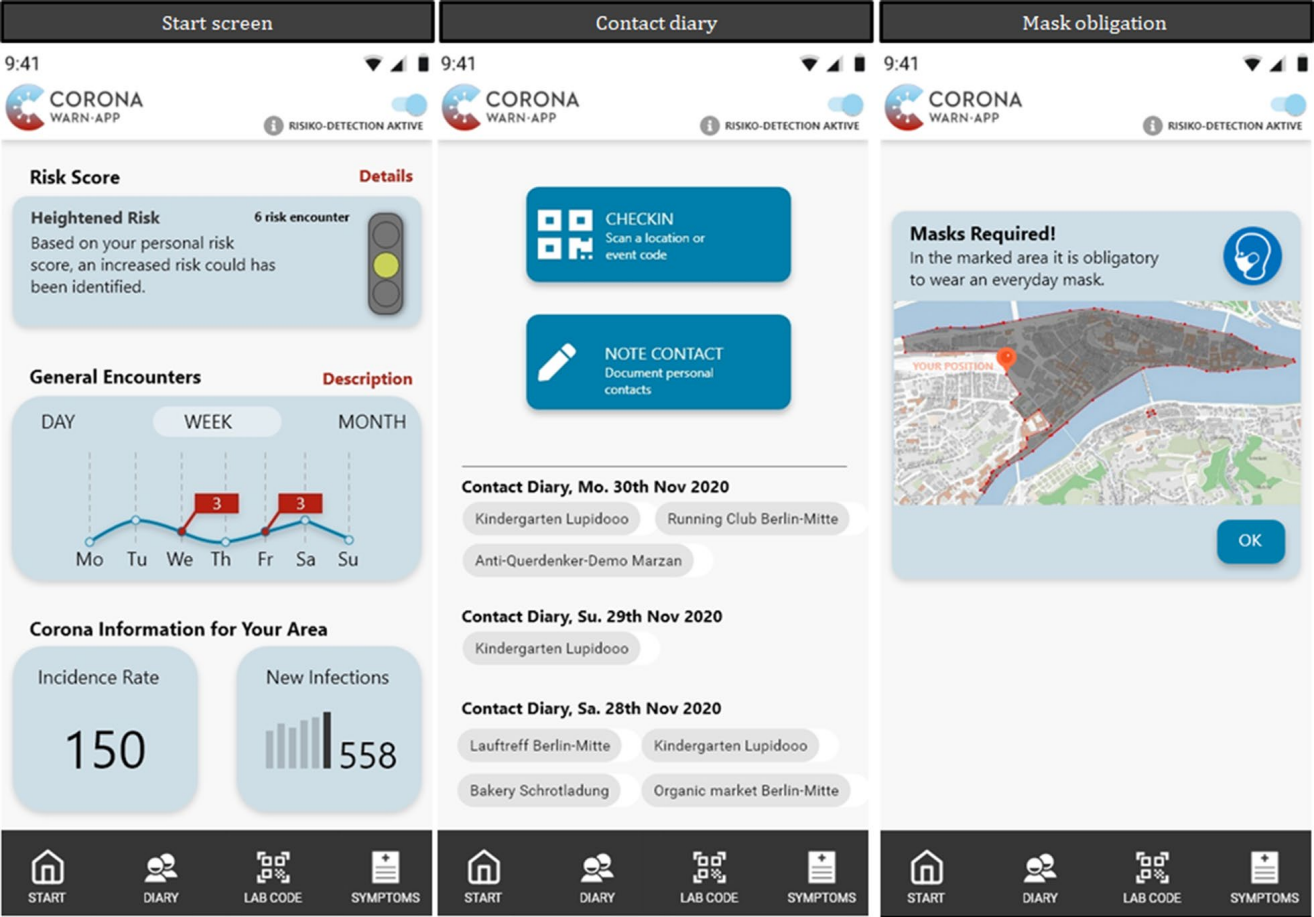
Selected screens of the enhanced CWA prototype^8^

Most of the user stories pertain to the front-end and are therefore implemented in our prototype to be tested within the third evaluation cycle. For better comparison reasons, we added earlier versions of the official CWA to the “Appendix” (cf. Fig. 9). Figure 4 presents an example of three screens. The first content row of the screen presents the user with additional details regarding the current risk assessment. Primarily, we added traffic-light logic. Second, the amount of risk contact is presented, and further details can be found with an information tap. In addition, the middle row on the primary screen displays a timeline with the general number of contacts and the risk contacts. Thus, users can initiate different actions, such as moving to the contact diary to check which personal contacts occurred. Meanwhile, the bottom row of the primary screen offers localized information about the coronavirus indicators. Due to its general publicity, this information shall be easy to understand and of great interest. The bottom navigation offers three options for further actions: a diary for contacts, a QR code scanner for the already-known test-center codes, and a novel feature for documenting a vaccination. Finally, symptom documentation has been integrated into our prototype. The contact diary (Screen 2) displays the already-documented contacts. To add an entry, we offer a check-in feature on the QR-code base; alternatively, the user might wish to write a manual entry. Furthermore, we added two features that are not visibly part of the app, itself: a push notification to the home screen warning the user about entering a geographical area with obligatory mask-wearing (Screen 3), and a second push notification informing the user about a public crowd ahead (according to E5, E7).

A crisis management app should include anon-German speaking people so that a preferred language can be selected. Regarding our prototype realization, we added an initial landing page, asking the user about their preferred language (between English or German). In addition, people with less digital affinity can use this app because all design elements are intuitive and self-explainable. Another important point to consider is people without a smartphone, which goes beyond the scope of this paper.

### Evaluation as part of the development

The extension of the CWA was developed within three extensive design cycles and evaluation cycles, having, in total, the following purposes: (1) acceptance, (2) additional functionalities, and (3) usability. In the following evaluation cycles’ sections, we include only the results, which can be transferred to the general field of crisis management. According to the FEDS framework [56], in the first iteration cycle, we conducted an ex-post, summative evaluation because we assessed the acceptance of the existing CWA with the help of 1992 German participants. The second and third iteration cycles are a formative evaluation for the development of our enhanced CWA prototype. Thereby, the second cycle was an ex-ante evaluation discussing with a focus group about which functionalities are valuable to integrate into the further German coronavirus tracing app. Based on this feedback, we developed the CWA prototype and evaluated it ex-post in a third evaluation cycle regarding usability. The evaluation of all three cycles was naturalistic because the pandemic situation is evident for each person, especially now during the second wave in Germany. Our evaluation can be attributed to the human risk and effectiveness strategy, despite the initial naturalistic cycle, because “a critical goal of the evaluation is to rigorously establish that the utility/benefit will continue in real situations and over the long run” [56]. As a result, in each iteration, the meta requirements (MR1–MR6) and design principles (DP1–DP3) were sharpened and refined.

#### Evaluation cycle 1: technology acceptance survey

This study was conducted approximately one month after launching the CWA in Germany. At this point, the use of the app occurred more passively (e.g., without coronavirus test results). The participants of the survey were asked about their attitudes and opinions concerning the current CWA. The age of the participants was distributed fairly normally. Most participants (1545) were between 25 and 54 years old. 1199 of the participants were male, 772 female, and 21 diverse. As summarized in the results from the TAM analysis of this survey (see Fig. 8, “Appendix”), the constructs perceived that usefulness, trust in technologies, and data concerns have a significant and noticeable influence on the behavioral intention to use the CWA. Furthermore, the descriptive results reveal interesting insights of users and their attitudes:

Overall, 97.9% of participants in our study were aware of the CWA. The onboarding channels that brought users to the app varied between the news (82.8%), taking the first position, followed by 54.9% general social media, followed by friends, family, and job at around 15%. Of these people, the vast majority (87.8%) stated that they were using the CWA. Six of those people felt forced to use the app (e.g., by employers or legal authorities), which is less than 0.5%. The main reason (96.6%) for using the app was the willingness of the respondents to participate in overcoming the crisis. Additionally, 61.2% wanted more clarity about the state of their health, and at least 24.4% wanted to support politics. Regarding the main reason for not using the app (12.2%), 36.8% of participants indicated data protection concerns, and 30.1% distrust the quality of the analysis. In nearly equal proportions (31%), the smartphone of our survey participants does not support the app, and even 29.3% claimed that they were annoyed by the very topic of the coronavirus.

In the “Appendix” (Table 4), 3 of 12 items stand out in the analysis of the descriptive results: usefulness, subjective norm, and privacy concerns. Considering the predominant use of the app by 87.8% of all study participants, this is remarkable due to the nearly low average of the perceived usefulness of 4.03 of 7 Likert points (LP). Thus, the assessment of the perceived usefulness reveals great potential for improvement. In the private context, people do not consider that performing their lives is more difficult without the CWA (1.93 LP). Additionally, most of the participants personally feel (5.88 LP) that their environment thinks (5.20 LP) that it is right to use the app. Protecting themselves is (rather) important to many participants, but it is much more important that they wish to protect their environment and not be responsible for infecting or quarantining others. Most participants simply wish to know as soon as possible if they are infected, which reveals that the risk calculation is consequently an important aspect.

Under the pandemic situation, there is an ongoing debate in academia and public interest groups concerning the potential privacy risks of tracing applications (e.g., [37, 52]). In this study, it became apparent that there are only a few concerns among users in the context of data transfer, but this point might be decisive for the behavioral intention to use the CWA. One reason for these fewer uncertainties might be the open-source approach—which emerged often within the free text fields—since it is possible to access what data is recorded and to whom it is provided. Within the free text fields, the participants shared much feedback (see Table 3), summarized in different issues (I1–I14 cf. Fig. 3).

**Table 3:** Feedback of the evaluation cycles

Evaluation cycle			Feedback
I	II	III	
X		X	More user involvement
X			Cross-border functionality within Europe
X			Participation in events should be possible, and the user should receive notifications
X	X		There must be a solution for people with older smartphone models
X		X	Connecting labs for all test results
X			More content information to lower data security concerns
X	X		At-work mode (e.g., for highly virus-protected health staff)
	X	X	General health application (e.g., gathering vital data, such as pulse, etc.)
	X		Vaccination integration
	X		Push notification for crowd sensitivity and mask obligation
	X		Create awareness for diseases
X	X	X	Overview about local coronavirus regulations
X	X	X	Overview about global coronavirus situation (e.g., infections)
		X	Capacity and utilization of test center
X		X	Details about risk contacts (also in graphical representation)
		X	Details about the meaning from when a risk contact has been hit
		X	Symptom tracking must have pre-formulated the most common symptoms and the degree of intensity
		X	Settings page in CWA: user must control the degree of data sharing

#### Evaluation cycle 2: usefulness and usability

As a main result of the first evaluation, the perceived usefulness should be enhanced. In a focus group featuring seven experts (see Table 1 for participants) as a second form of evaluation, we discussed existing and novel functionalities based on the meanings of experts from online news and practical literature, such as from epidemiologists (see “Multi-methodological approach” section for more details). This focus group workshop was structured as follows: In the beginning, (1) we provided a ranking of the identified functions that were collected during the first iteration cycle. Then, (2) the experts discussed the overall ranking and added further functional requirements that they proclaimed to be relevant. (3) This was followed by another round of ranking, and finally, (4) we discussed the future use of the CWA. In total, we added three new functions to our function set (see Table 2), as we have not found these in the white and practical literature analysis or in the users’ feedback.

A point of discussion within the group of experts was the data protection aspect, but in terms of developing a more reasonable attitude, data protection cannot always be the knockout argument, particularly not during a pandemic situation. There is a need for a pandemic instrument for society to not be forced to repeatedly enter lockdowns, since the virus will surely return in waves until herd immunity is reached (E4, E6). Therefore, a suitable IT instrument in crisis management must focus on future possibilities, such as vaccination used as permission to travel to other countries (E4). Another possibility is that the tracing app will be extended to an overall health app by integrating vital parameters, such as heart rate, in terms of an app for prevention (E2).

As a result of the focus group, the main purpose of the CWA should be about creating awareness (E7) and, thus, presenting the local coronavirus regulations (e.g., limitations of contacts) (E5), how many people are being met (E4), and the presence of clusters in surrounding areas [37, 42, 43]; E7]. It is important to obtain more details about an infected contact person to estimate the risk of coronavirus disease 2019 infection, such as for people in high-risk environments [29]; E4]. In the case of crowding or mask obligation, users should be informed via push notifications (E5). Moreover, the test results were ranked second so that the connectivity to all labs should work.

#### Evaluation cycle III: usability

**Fig. 5:**
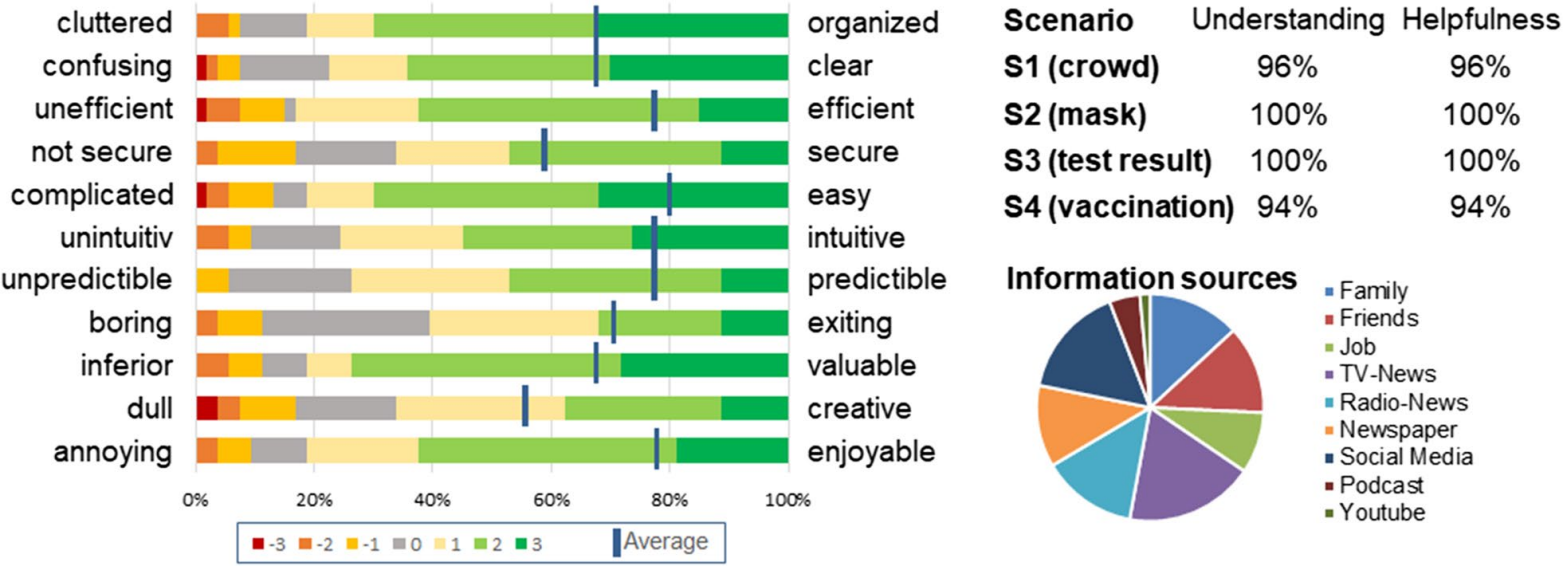
Results of the prototype evaluation

The third evaluation cycle was performed through an online survey combined with testing our prototype, including our feature and design enhancements. The structure and results of the survey are presented in Table 5 (“Appendix”). At the beginning of this survey, the focus was on testing the full prototype, partly depicted in Fig. 4, and assessing the usability. Then, four different scenarios^7^ were tested: crowding, mask obligation, vaccination, and coronavirus testing. At the end, final questions were asked concerning demographics and to again compare the usefulness of the current CWA (as in the first evaluation cycle) and the extended version. The most important results of the questionnaire are presented in Fig. 5.

For all usability items according to Schrepp et al. [49], at least 60% of the participants positively assessed our prototype (5–7 LP, see Fig. 5 on the left side). The respondents understood all four presented scenarios and found them to be helpful (see Fig. 5 on the upper right side). In addition, the respondents were asked about sources of information for pandemic content, which has already lasted 10 months. Television news was indicated by 71.70% of participants as being the most used source of information for remaining up to date regarding the coronavirus, but this was closely followed by 62.26% of participants who used social media (see Fig. 5 on the bottom right side). All respondents saved their coronavirus test results in the app, and only 1.89% would not share their results with their contacts via the app (1–3 LP). Among 39.62% of participants who have already been tested, 76.19% reported sending a positive test to their met people and health authorities using the app. Regarding vaccination, the participants are similarly open-minded: 94.34% currently save their coronavirus vaccination in the app, and 90.57% share this information.

The majority seek more details regarding risk contacts (4.83 LP), whereby they would be willing to accept more data protection restrictions for this purpose (5.23 LP). The respondents perceive there to be insufficient usefulness in the current CWA (4.27 LP), though 86.79% use it. The assessment of usefulness has increased significantly regarding our extension (6.04 LP), and the participants would use it in the future (5.94 LP). Opinions of the respondents regarding vital signs are mixed but rather averse (3.83 LP). Summarized from all three design cycle iterations, we gathered valuable feedback, presented in Table 3.

## Discussion and implications

Our study provides implications on the application level for COVID-19 tracing apps, such as the CWA. From a broader perspective, we can derive learnings for an iterative design approach—as presented in this research—in the context of crisis management and pandemic countermeasures. We believe that this perspective matters substantially under the predictable continuation of global pandemic diseases but even further for the design of digital crisis management artifacts of any kind, whether refugee, climate, or any other global crisis.

### Implications for practice in digital responses to pandemic apps

Our paper provides both scientific and practical implications. First, we assembled a set of issues (I1–I14) with the current CWA, which we argumentatively deductively transformed into meta-requirements (MR1–MR6) for the CWA. Based on this, we developed design principles (DP1–DP3) that can be used as a foundation for the further development of COVID-19 tracing apps and pandemic apps in general. Moreover, our artifacts can serve as starting points for further in-depth research. Our proposed changes would significantly improve the perceived usefulness of pandemic apps, based on the survey results (see Tables 4, 5, “Appendix”). We assume that either the usefulness has been poorly communicated to the broad public and/or the advantage has not yet been present in its entirety thus far through the provision of desired features. This assumption is strengthened by the fact that a variety of academic and private initiatives is investigating reliable indicators concerning the app usage and the reported (approved) infections (e.g., [5]. Recent research questions the validity of the Corona-Warn-App (and other COVID-19 tracing applications) approach because evidence is lacking regarding the effectiveness of such apps and their pandemic containment effect. For instance, Leith and Farrel [34] state that the proximity detection of, for instance, the German COVID-19 tracing app might not capture any contact when using a tram. Instead, the Italian configuration of the same framework generates significantly more contacts (for the same experimental scenario). Overall, these facts might explain our finding of the limited perceived usefulness.

The development process of the CWA can be categorized more as classical requirement engineering and less as agile development practices. The latter appears to be implemented for the core development team (following their activities on GitHub), but the RKI and the German Ministry of Health and the Federal Chancellery do not follow an agile approach in their application management. Thus, classical change and release management appear to dominate their mindset. For a highly dynamic environment, such as the ongoing pandemic and multiple waves of infections, software development must deal with moving targets. Agile software management practices might do a better job in such a context than can traditional approaches. We argue that IS shall further embrace agile practices for scholars and researchers. Furthermore, our findings strengthen a governmental, yet agile, development approach: While epidemiologists provide recent research results to policymakers to balance interventions and human rights, it is in the hands of the information system researcher to generate learnings on the digital COVID-19 containment options (such as the CWA) and pandemic-related apps in general. However, scientists are in competition with economically motivated projects (such as the German Luca-App), which lack privacy-by-design [51] but create much attention and public acceptance when better meeting requested user needs than the governmental app. The risk that occurs here is that the government loses its direct communication line through the official pandemic app and becomes a minor, less popular niche application.

Our findings reveal the positive attitude of users and health experts regarding voluntary data donations within the CWA. This is congruent to the most recent recommendation of the largest scientific advisory council in Germany, Leopoldina [35]. At the time of submitting this paper, the growth rate of new infections is (still) slowly growing; the epidemiological researchers of the Leopoldina recommend a strict lockdown and an extension of the CWA (e.g., “data donations”) [35], p. 5]. Regarding our aforementioned findings of improved digital connectivity of laboratories and other health-related government authorities, they continue, “Efforts to digitize infection control should be intensified. It is important to especially have adequate digital equipment for the public health service” [35], p. 5]. Moreover, they stress the point of user-centric communication and recommend much more use and intensity of explanatory content communication, which is implemented in our prototype.

If pandemic control is not successful, then, as leading epidemiologists suspect, further lockdowns are unavoidable. These considerations should be considered in further developments of the pandemic apps. For example, the app could indicate testing and vaccination centers and offer a booking of those appointments. In addition, a detailed discussion should be held about whether it is meaningful to leave the decision of sharing (or not sharing) the test result to the users. In our view, the option of not sharing test results is counterproductive. The rationale behind this is that non-pharmaceutical interventions on public life will last longer or possibly be exacerbated. In terms of balancing data privacy against high death rates and negative economic impact, the authors of this study argue for the automatic, anonymized sharing of test results. Furthermore, the evaluation cycles (especially cycles one and three) indicate that the use of a wide variety of communication channels is sensible. In this way, different user groups can be addressed.

**Fig. 6:**
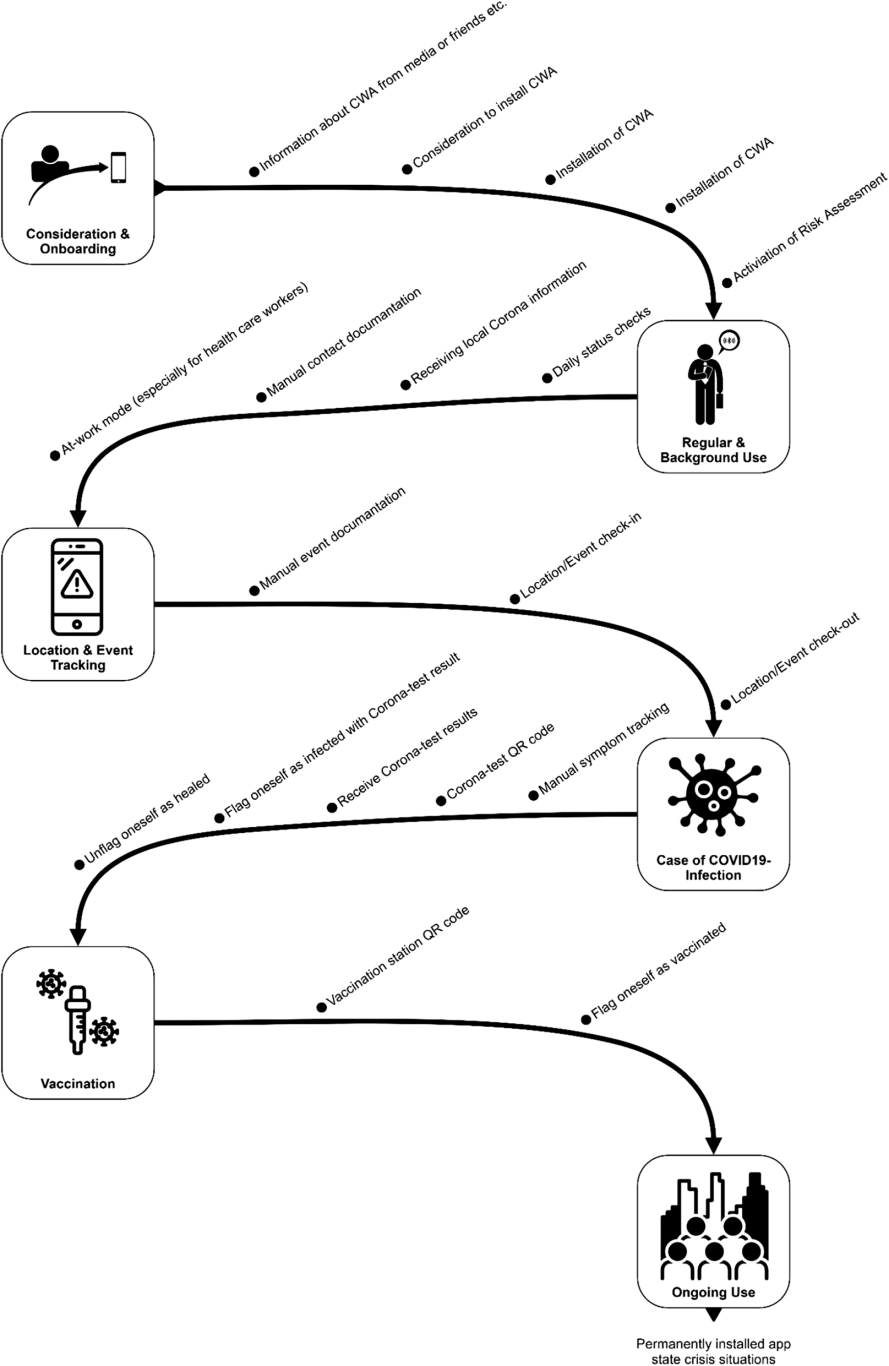
Enhanced user journey of the CWA

**Fig. 7:**
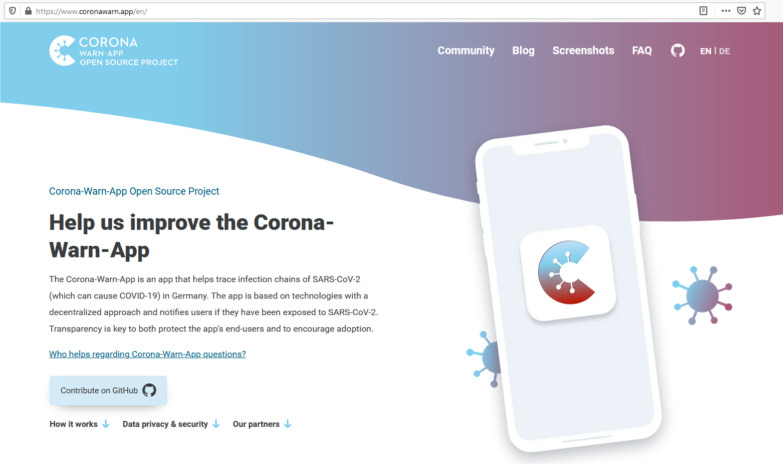
Call for participation on the official CWA website

**Fig. 8:**
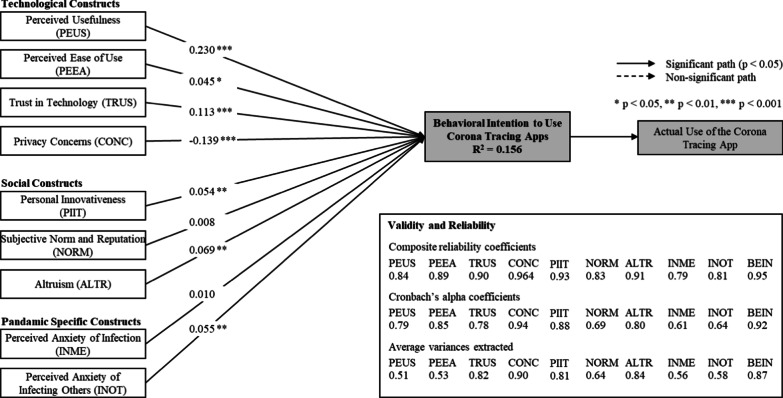
Results of TAM analysis using PLS-SEM (first evaluation cycle) [31]

**Fig. 9:**
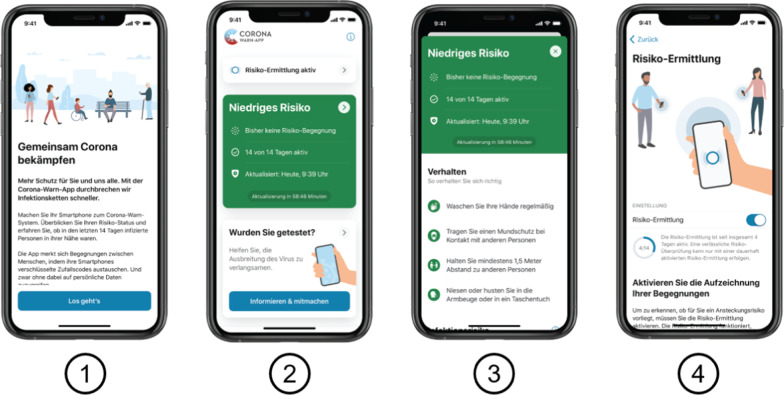
Original CWA-website screens provided only in German (Robert-Koch-Institut 2020, cf. https://github.com/corona-warn-app/cwa-documentation/blob/master/images/ui_screens/ui_screens_ios.png. Accessed 18 April 2021)

As the Google/Apple framework fully represents the current feature set of the CWA, the RKI must discuss the USP (and therefore the reasons why to use the app, tax investments, and so on). The second to release version 1.9 of the CWA^11^ is even more irritating for the user, as multiple low-risk contacts (marked as green inside the app) can accumulate to become a high-risk score (marked in red). Furthermore, the number of risk contacts the CWA displays in the freshly deployed version is instead the number of days with risk contacts,^12^ which still does not provide any clear indication to the user where or when contact took place. Instead, the user experience changes radically without—compared with the proposals developed in our paper—any user-centered benefit. However, it can be positively interpreted that the CWA has faced slight enhancements in the second wave, and even ideas from this paper were included. The most recent update, rolling out during the submission of this paper, will provide a basic diary functionality for places and people met.^13^ Nevertheless, regarding the development of pandemic apps, we recommend a pandemic-related view into the entire user journey (cf. Fig. 6), to address the existing crisis holistically, instead of implementing micro-features. Furthermore, there are more functions, which should be concerned, developing a pandemic app.

Finally, we present an updated version of the German COVID-19 tracing app user journey as a comprehensive artifact in Fig. 6. In addition to the presented and discussed essential steps from the previous section, we include the suggestion from evaluation cycle 2 a bit further by recommending the German COVID-19 tracing app as a potential replacement for the existing NINA app to the Federal Office of Civil Protection and Disaster Assistance.^14^ Instead of having multiple crisis apps in Germany, in the long run, the CWA could be renamed as a more general warning and disaster assistance application and combine the pandemic and other crisis warning use cases. Thus, such permanent installation and regular use could offer enhancements, and a direct crisis communication line to the German public would exist. From a broader perspective, this artifact might be seen as a blueprint for other privacy-preserving COVID-19 tracing apps that build upon the Google/Apple framework.

### Implications for research in crisis management

This article provides several implications at the international level for the fields of emergency management, crisis communication, and crisis management. The newly designed example of the German COVID-19 tracing app represents a novel approach of voluntary involvement of the population via open-source (GitHub) in crisis management; thus, the development process of this technology was unique, as well. For health institutions on a global (WHO) or national level, we recommend a design-science-oriented approach such as the one adopted in this paper. DSR approaches provide well-elaborated and trusted iteration and evaluation cycles. By identifying meta-requirements (MR1–MR6) and design principles (DP1–DP3), we provide further insights into science. According to Gregor and Hevner [15], design principles contribute to nascent design theory. This knowledge can be leveraged to develop similar applications in other crises, for instance.

The wide response to our first survey with 1,992 participants for Germany alone clearly demonstrates the high interest in participatory development. As trust matters significantly for the mass acceptance of crisis technology [23], this factor should be emphasized by governments regarding more open source development. Comparing the results of our first to the final iteration, the differences in the scope of the technology become visible. Thus, involving different types of users and experts appears to be a promising pattern under the development of a crisis, such as the ongoing pandemic. Through that, biases and other limitations by a singular perspective onto a problem might be avoidable at higher chances. Regarding the significant crises we are facing—COVID-19, refugee, and climate crises—an iterative, highly flexible design and engineering of crisis management tools of any kind appear to be recommendable. Only such an approach inherits the flexibility in the engineering process to deal with a moving target. Moreover, young research underlines this argument through studies, indicating the resistance of people to install more than one governmental crisis app [25] and the recent development in Germany regarding a start-up-based coronavirus application, which some people understood to be an alternative to the CWA, to strengthen our argumentation for a well-designed, mass-acceptance-oriented governmental app-building approach accompanied by research [51].

A further observation and implication of this paper is the fact that neither government institutions nor the WHO developed a solution for COVID-19 applications; rather, Google and Apple surprisingly announced and deployed a de facto technical standard-binding framework. The implicit learning of this reveals that governments must be able to provide a counterbalance in requirement specification. Therefore, our approach is feasible to guide policymakers throughout the development process of an ad hoc crisis management technology.

## Conclusion and outlook

In this study, we present concrete enhancements for the German Corona-Warn-App transferable to other pandemic apps, based on an iterative DSR approach, and derived further learnings for crisis management applications. Throughout three rounds of iterations and evaluation, we presented, prototyped, and tested user-centered ideas of improvement for increasing the perceived usefulness of the CWA.

Our findings suggest a strict agile development pipeline. The CWA began from an application engineering perspective, which is today completely solved by the operating system and the ENF. Indeed, the entire user journey appears in a completely different picture during the current second pandemic wave. While the current development team appears to already work in agile settings, we further recommend an agile and therefore ongoing understanding of the German COVID-19 tracing application manager role (i.e., Scrum Product Owner instead of a reporting- and documentation-oriented manager). Thus, it might be recommendable for the involved decision-makers and stakeholders to switch from classic application management to an agile setup, which allows for more flexible reaction to upcoming changes. This argument is strengthened by our data from medical experts acquired in the second evaluation cycle, in which our research shall create the sensitivity in science, public, and government institutions that this pandemic will neither be over soon nor be the last one, as history has demonstrated.

As with every study, this paper is limited by multiple factors. The current situation makes it almost ethically and practically impossible to include physicians in the IS research. Using highly flexible scheduling and digital scheduling tools, we attempted to include experts from the broader health sector regarding shifting hours. Although we appreciate the size and in-depth quality and experience of the achieved focus group, one could argue that such a setting might be biased. Furthermore, as this research is part of a longer DSR project, the initial study was focused worldwide and localized only for Germany. Thus, the primary sample is independent of the third sample of participants for the online survey combined with testing our prototype.

While this paper offers clear recommendations for the feature set of the CWA and some slight UI/UX enhancements, future research might focus on interaction or even gamification aspects to increase the user interaction inside the app.

In all, we must be mindful that the current pandemic situation will not be the last crisis. Thus, it is even more important for governments to have a well-established, flexible, design-oriented process for creating and adapting technology to handle a crisis.

### Supplementary Information

**Additional file 1**. Survey structures and interview guideline.

## Data Availability

The datasets generated and analyzed during the current study are available here (repository of the University Osnabrueck): https://myshare.uni-osnabrueck.de/d/6e8a42d535d648cfbef6/ (please also see section Supplementary information). The derived results can be find in this article.

## Appendix

See Tables 4, 5 and Figs. 7, 8, 9).

**Table 4:** Survey structure and results of evaluation cycle III

Construct	Item	Values	Average	Median	SD
Behavioural intention	I would consider to share data with the official corona tracing app	1–7	5.49	6.00	1.86
	I would be willing to share data with the official corona tracing app	1–7	5.47	6.00	1.84
	I would like to share data with the official corona tracing app	1–7	5.00	6.00	2.02
Perceived usefulness	I think I have a better possibility to visit business or social events using the corona tracing app (workshops, restaurants, clubs, sport, etc.)	1–7	3.53	4.00	1.92
	My private life would be difficult to perform without the corona tracing app	1–7	1.93	1.00	1.27
	My job would be difficult to perform without the corona tracing app	1–7	1.85	1.00	1.27
	Using the corona tracing app makes it easier to cope with the pandemic situation	1–7	5.39	6.00	1.61
	Using the corona tracing app gives me greater control over my health	1–7	4.11	5.00	1.75
	The corona tracing app addresses my expectations	1–7	5.34	6.00	1.50
	Overall, I find the corona tracing app useful	1–7	5.79	6.00	1.50
	I feel more safe using the corona tracing app	1–7	4.33	5.00	1.74
Perceived ease of use	I found it easy to install the corona tracing app	1–7	6.69	7.00	0.70
	I often become confused when I use the corona tracing app	1–7	1.70	1.00	1.10
	I often get errors when using the corona tracing app	1–7	2.03	1.00	1.61
	Interacting with the corona tracing app is often frustrating	1–7	1.62	1.00	1.05
	The corona tracing app often behaves in unexpected ways	1–7	1.62	1.00	1.09
	My interaction with the corona tracing app is easy for me to understand	1–7	6.27	7.00	1.03
	I like the design of the corona tracing app	1–7	5.42	6.00	1.20
	Overall, I find the corona tracing app easy to use	1–7	6.33	7.00	0.84
	I found it easy to follow instructions or recommendation of the corona tracing app	1–7	6.25	6.00	0.43
	The exchange between the health department and the corona tracing app was easy	1–7	5.25	5.00	1.30
Trust	I usually trust a technology until it gives me a reason not to trust it	1–7	4.31	5.00	1.72
	I generally give a technology the benefit of the doubt when I first use it	1–7	4.29	5.00	1.58
	The corona tracing app cannot be trusted, there are just too many uncertainties	1–7	1.93	1.00	1.36
Privacy concerns	How much are you concerned that the information submitted to the corona tracing app can be used in a way you did not foresee	1–5	1.78	1.00	1.12
	How much are you concerned that the information submitted to the corona tracing app can become available to someone you don’t want	1–5	1.87	1.00	1.17
	How much are you concerned that the information submitted to the corona tracing app can become available to someone without your knowledge	1–5	1.91	2.00	1.18
Subjective norm	Using the corona tracing app is ‘the right thing to do’	1–7	5.88	6.00	1.48
	Using the corona tracing app sets a good example	1–7	5.77	6.00	1.58
	My family or friends think it's a good thing using the corona tracing app	1–7	5.20	5.00	1.43
Reputation	I earn respect from others by actively participating in the corona tracing app	1–7	3.15	4.00	1.53
	I feel that active participation improves my status	1–7	2.98	3.00	1.60
Personal innovative ness	If I heard about a new information in the domain of technology, I would look for ways to experiment with it	1–7	4.71	5.00	1.55
	Among my family and friends, I am usually the first to try out new information technologies	1–7	4.58	5.00	1.81
	In general, I am sceptical to try out new information technologies	1–7	2.96	3.00	1.53
	I like to experiment with new information technology	1–7	4.92	5.00	1.60
Altruism	I enjoy using technologies if it benefits others	1–7	5.60	6.00	1.19
	I enjoy helping others	1–7	6.03	6.00	0.87
	It feels good to help someone else	1–7	6.19	6.00	0.85
Fearing the unknown	When I don't know what's happening, I feel uncomfortable or stressed	1–7	4.92	5.00	1.29
	It frustrates me not having all the information I need	1–7	5.33	5.00	1.23
	I prefer to plan everything in my life	1–7	4.49	5.00	1.45
	I am afraid of receiving an infection warning without knowing further details (contact, time, place)	1–7	3.34	3.00	1.67
Anxiety of infection	I am afraid of getting infected with COVID-19	1–7	4.38	5.00	1.66
	I want to know my personal risk of being infected (without the need of a classical COVID-19 test)	1–7	5.01	5.00	1.46
	I want to know a potential infection as soon as possible	1–7	6.03	6.00	1.29
	I don't want to be in quarantine	1–7	5.06	5.00	1.71
Anxiety to infect other	I am afraid of infecting others with COVID-19	1–7	5.65	6.00	1.50
	I want to inform automatically others in case that I am infected	1–7	6.14	7.00	1.31
	I do not want to be responsible infecting others	1–7	6.39	7.00	1.00
	I do not want to be responsible for the quarantine of others	1–7	5.87	6.00	1.42

*SD* standard deviation

**Table 5:** Survey structure for evaluation cycle III (SD = Standard Deviation, NA = No Answer)

Item	Values	Average	Median	SD
Question group: an enhancement of the Corona-Warn-App				
Usability				
Unpleasant–pleasant	1–7	5.49	6	1.28
Dull–creative	1–7	4.87	5	1.49
Inferior–valuable	1–7	5.66	6	1.41
Boring–exciting	1–7	4.89	5	1.27
Unpredictable–predictable	1–7	5.26	5	1.09
Unintuitiv–intuitiv	1–7	5.42	6	1.42
Complicated–simple	1–7	5.62	6	1.52
Unsafe–safe	1–7	5.04	5	1.37
Inefficient–effective	1–7	5.36	6	1.46
Confusing–clear	1–7	5.58	6	1.42
Cluttered–tidy	1–7	5.70	6	1.38
Is the level of detail for risk contacts sufficient?	1–7	5.19	6	1.24
Would you prefer to get more details about a risk contact?	1–7	4.83	5	1.72
Would you be willing to accept privacy restrictions yourself for more details about risk contacts?	1–7	5.23	6	1.67
Question group: Scenario 1 Crowd of people		Yes	No	NA
Did you understand the feature?	Yes/No/NA	96.23%	3.77%	0%
Did you find the feature helpful?	Yes/No/NA	83.02%	17.88%	0%
Question group: Scenario 2 Mask obligation		Yes	No	NA
Did you understand the feature?	Yes/No/NA	100.00%	0%	0%
Did you find the feature helpful?	Yes/No/NA	90.57%	9.43%	0%
So, understanding how GPS coordinates can help you perform other functions of the app, would you accept the recording of your GPS coordinates?	Yes/No/NA	83.02%	11.32%	5.66%
Question group: Scenario 3 Corona test		Yes	No	NA
Did you understand the feature?	Yes/No/NA	100.00%	0%	0%
Would you be willing to save your test in the app?	Yes/No/NA	100.00%	0%	0%
Would you share your test with your contacts and health authorities?	Yes/No/NA	94.34%	1.89%	3.77%
In the following, you can share your reasons for not wanting to share your Corona test with contacts or health authorities through the app		Free text field		
Have you already been tested during the pandemic?	Yes/No/NA	39.62%	60.38%	0%
Regarding your test: Did you receive the test result via in your app?	Yes/No/NA	42.86%	57.14%	0%
Did you share the test result to met people via the app (or in case of a negative test: would you have shared a positive test result)?	Yes/No/NA	76.19%	23.81%	0%
Question group: Scenario 4 Vaccination		Yes	No	NA
Did you understand the feature?	Yes/No/NA	94.34%	3.77%	1.89%
Would you be willing to save your vaccination in the app?	Yes/No/NA	94.34%	3.77%	1.89%
Would you share it with health authorities?	Yes/No/NA	90.57%	5.66%	3.77%
In the following, you can share your reasons for not wanting to share your vaccination with health authorities through the app		Free text field		
Question group: Personal and closing questions		Average	Median	SD
How old are you?	18–24, 25–34, 35–44, 45–54, 55–64, 65–74 years old, older than 74	29.95	29.5	9.14
		Yes	No	NA
Do you own a smartphone?	Yes/No/NA	100.00%	0%	0%
Do you use the current Corona-Warn-App?	Yes/No/NA	86.79%	9.43%	− 3.78%
		Average	Median	SD
Do you find the current Corona-Warn-App useful?	1–7	4.27	5	1.27
Please answer the following questions regarding the advanced Corona-Warn-App presented here … the extension of the app is useful	1–7	6.04	6	1.18
… the service provider of this App is trustworthy	1–7	5.58	6	1.23
… sharing my data with the Corona-Warn-App involves a high privacy risk for me	1–7	3.36	3	1.78
… in the future, I would use the Corona-Warn-App if it includes the features presented here	1–7	5.94	6	1.41
… I would record other vital parameters (pulse. stress level. etc.) in the Corona-Warn-App if this would create additional functionalities	1–7	3.83	4	1.86
		Yes	No	
What sources of information do you use to stay up to date on the pandemic?	Family	50.94%	49.06%	
	Friends	49.06%	50.94%	
	Job	33.96%	66.04%	
	TV news	71.70%	29.30%	
	Radio news	52.83%	47.17%	
	Newspaper	45.28%	54.72%	
	Social media	62.26%	37.74%	
	Podcast	16.98%	83.02%	
	Youtube	5.66%	94.34%	
Do you have further suggestions for improving the prototype?		Free text field		

## Abbreviations

CWA: Corona-Warn-App
DP: Design principle
DSR: Design science research
ENF: Exposure notification frameworks
LP: Likert points
MR: Meta-requirements
PLS-SEM: Partial least squares structural equation modeling
TAM: Technology acceptance model

## Footnotes

1 WHO Coronavirus (COVID-19) Dashboard, see https://covid19.who.int/. Accessed 18 April 2021.

2 See https://developer.apple.com/documentation/exposurenotification/enexposureconfiguration. Accessed 17 Dec 2020.

3 RKI stands for Robert Koch Institute, which is the central institution of the German Federal Government in the field of disease surveillance and prevention, see https://www.rki.de/, accessed 18 April 2021

4 See https://www.rki.de/DE/Content/Service/Stellen/Angebote/2020/101_20.html. Accessed 9 Dec 2020.

5 See https://www.coronawarn.app/en/. accessed 18 April 2021, see Fig. 7 in “Appendix”.

6 See https://github.com/corona-warn-app/cwa-documentation/blob/master/user_journey.png. Accessed 20 Sep 2020.

7 SAP is a large publicly traded software company commissioned to develop the CWA. For more information about SAP, see https://www.sap.com/index.html

8 See https://www.coronawarn.app/de/faq/#recovered. Accessed 8 Dec 2020.

9 See https://github.com/corona-warn-app/cwa-documentation/blob/master/cwa-risk-assessment.md. Accessed 11 Dec 2012.

10 The prototype is accessible via https://t1p.de/bisecwa-en (English version). and https://t1p.de/bisecwa (German version). We also provide a demo video: https://t1p.de/wzu8.

11 See deployed version 1.9 of the Corona-Warn-App: https://www.coronawarn.app/de/blog/2020-12-17-risk-calculation-exposure-notification-framework-2-0/. Accessed 17 Dec 2020.

12 See https://www.coronawarn.app/de/blog/2020-12-16-corona-warn-app-version-1-9/. Accessed 17 Dec 2020.

13 See https://www.coronawarn.app/de/blog/2020-12-28-corona-warn-app-version-1-10/. Accessed 04 Jan 2021.

14 BBK: Warn-App Nina, see https://www.bbk.bund.de/EN/Home/home_node.html;jsessionid=5B59D830FADE9AF5696BEF0B9C5E652A.2_cid509. Accessed 19 Dec 2020.
