# Supplementary material for: Learnings from the design and acceptance of the German COVID-19 tracing app for IS-driven crisis management: a design science research
Source: BMC Med Inform Decis Mak. 2021 Aug 9;21:238. doi: 10.1186/s12911-021-01579-7 (PMC8350273; doi:10.1186/s12911-021-01579-7)
Supplement: Supplementary file 1 — Additional file 1. Survey structures and interview guideline. [file 12911_2021_1579_MOESM1_ESM.pdf]

Learnings from the Design and Acceptance of the German COVID-19 Tracing App for IS-driven Crisis Management: A Design Science Research

Supplementary file: Survey structures and interview guideline

Scales:

Please indicate the degree of your agreement.<br>1 = Strongly disagree<br>2 = Disagree<br>3 = Rather disagree than agree<br>4 = Neither disagree or agree<br>5 = Rather agree than disagree<br>6 = Agree<br>7 = Strongly agree	1 = Not at all concerned<br>2= Slightly concerned<br>3 = Somewhat concerned<br>4 = Moderately concerned<br>5 = Extremely concerned

Survey of evaluation cycle I

1. Do you know the official corona tracing app of your country?
2. How did you know about the app? (Answers: Family, Friends, Job, News, Social Media, Others: __)
3. Do you use the official corona tracing app?
4. Are you forced (i. e. by legal authorities, employer, etc.) to use the app?
5. Why do you use this app? (you can choose more than one answer: Contribute to overcoming the crisis, More clarity about my own health, social acknowledgement, political support, people from my environment use this app, Others: _______)
6. Why do you not use this app? (you can choose more than one answer: time or organizational effort of app installation, no support of my smartphone, data protection concerns/possibility of data misuse, mistrust in technologies, mistrust in the quality of analyses, annoyed by corona issue, sensitivity of data, Others: _______)
7. Did the official corona tracing app ask you to contact your doctor or public health department so far?
8.
   a. I would consider to share data with the official corona tracing app.
   b. I would be willing to share data with the official corona tracing app.
   c. I would like to share data with the official corona tracing app.
9.
   a. I think I have a better possibility to visit business or social events using the corona tracing app (workshops, restaurants, clubs, sport etc.).
   b. My private life would be difficult to perform without the corona tracing app.
   c. My job would be difficult to perform without the corona tracing app.
   d. Using the corona tracing app makes it easier to cope with the pandemic situation.
   e. Using the corona tracing app me greater control over my health.
   f. The corona tracing app addresses my expectations.
   g. Overall, I find the corona tracing app useful.
   h. I feel more safe using the corona tracing app.
10.
   a. I think I have a better possibility to visit business or social events using the corona tracing app (workshops, restaurants, clubs, sport etc.).
   b. My private life would be difficult to perform without the corona tracing app.
   c. My job would be difficult to perform without the corona tracing app.
   d. Using the corona tracing app makes it easier to cope with the pandemic situation.
   e. Using the corona tracing app me greater control over my health.
   f. The corona tracing app addresses my expectations.
   g. Overall, I find the corona tracing app useful.
   h. I feel more safe using the corona tracing app.
11.
   a. I often become confused when I use the corona tracing app.
   b. I often get errors when using the corona tracing app.
   c. I found it easy to install the corona tracing app.
   d. Interacting with the corona tracing app is often frustrating.
   e. The corona tracing app often behaves in unexpected ways.
   f. My interaction with the corona tracing app is easy for me to understand.
   g. I like the design of the corona tracing app.

h. Overall, I find the corona tracing app easy to use.
i. I found it easy to follow instructions or recommendation of the corona tracing app.
j. The exchange between the health department and the corona tracing app was easy.

12.
a. I usually trust a technology until it gives me a reason not to trust it.
b. I generally give a technology the benefit of the doubt when I first use it.
c. The corona tracing app cannot be trusted, there are just too many uncertainties.

13. How much are you concerned that the information submitted to the corona tracing app:
a. ...can be used in a way you did not foresee.
b. ...can become available to someone you don't want.
c. ...can become available to someone without your knowledge.

14.
a. Using the corona tracing app is 'the right thing to do'.
b. Using the corona tracing app sets a good example.
c. My family or friends think it's a good thing using the corona tracing app.
d. I earn respect from others by actively participating in the corona tracing app.
e. I feel that active participation improves my status.

15.
a. If I heard about a new information in the domain of technology, I would look for ways to experiment with it.
b. Among my family and friends, I am usually the first to try out new information technologies.
c. In general, I am sceptical to try out new information technologies.
d. I like to experiment with new information technology.

16.
a. I enjoy using technologies if it benefits others. (Cropanzano and Mitchell 2005)
b. I enjoy helping others. (Cropanzano and Mitchell 2005)
c. It feels good to help someone else.
d. I'm usually aware of my health.
e. I take responsibility for the state of my health.
f. I listen to my body feelings when I go through the day.

17.
a. When I don't know what's happening, I feel uncomfortable or stressed.
b. It frustrates me not having all the information I need.
c. I prefer to plan everything in my life.
d. I am afraid of receiving an infection warning without. knowing further details (contact, time, place).

18.
a. I am afraid of getting infected with COVID-19.
b. I want to know my personal risk of being infected (without the need of a classical COVID-19 test).
c. I want to know a potential infection as soon as possible.
d. I don't want to be in quarantine.

19.
a. I am afraid of infecting others with COVID-19.
b. I want to inform automatically others in case that I am infected.
c. I do not want to be responsible infecting others.
d. I do not want to be responsible for the quarantine of others.

20.
a. Is there anything else you want to share with us regarding this topic?
b. Was a person close to you infected by COVID-19? (Answers: family member, friend, colleague, relative of a friend, Other: ____)
c. Age and Gender
d. Country
e. What describes best the area where you live (population)?
    i. Village (<5,000)
    ii. Small town (>5,000 up to 20,000)
    iii. Medium city (>20,000 up to 100,000)
    iv. Large city (>100,000 up to 500,000)
    v. Metropolis (more than 500,000)

Guidance of the interview in evaluation cycle II

1. Introduction of all participants
2. How can you relate to the pandemic?
3. How to improve the Corona-Warn-App functionally?
    a. What functions should be integrated?
    b. What can we learn from COVID-19 apps in other countries?
    c. What about the functional, technical connectivity of offices and labs?
    d. Do you have any ideas for the future of COVID-19 apps?
4. Ranking of identified functions for an enhanced Corona-Warn-App (2 runs).

Survey of evaluation cycle III

**Item**		**Values**
Question group: An enhancement of the Corona-Warn-App		
Usability	unpleasant - pleasant	1-7
	dull - creative	1-7
	inferior - valuable	1-7
	boring - exciting	1-7
	unpredictable - predictable	1-7
	unintuitiv - intuitiv	1-7
	complicated - simple	1-7
	unsafe - safe	1-7
	inefficient - effective	1-7
	confusing - clear	1-7
	cluttered - tidy	1-7
Is the level of detail for risk contacts sufficient?		1-7
Would you prefer to get more details about a risk contact?		1-7
Would you be willing to accept privacy restrictions yourself for more details about risk contacts?		1-7
Question group: Scenario 1 Crowd of people		
Did you understand the feature?		Yes/No/NA
Did you find the feature helpful?		Yes/No/NA
Question group: Scenario 2 Mask obligation		
Did you understand the feature?		Yes/No/NA
Did you find the feature helpful?		Yes/No/NA
So, understanding how GPS coordinates can help you perform other functions of the app, would you accept the recording of your GPS coordinates?		Yes/No/NA
Question group: Scenario 3 Corona test		
Did you understand the feature?		Yes/No/NA
Would you be willing to save your test in the app?		Yes/No/NA
Would you share your test with your contacts and health authorities?		Yes/No/NA
In the following, you can share your reasons for not wanting to share your Corona test with contacts or health authorities through the app.		
Have you already been tested during the pandemic?		Yes/No/NA
Regarding your test:<br>Did you receive the test result via in your app?		Yes/No/NA
Did you share the test result to met people via the app (or in case of a negative test: would you have shared a positive test result)?		Yes/No/NA
Question group: Scenario 4 Vaccination		
Did you understand the feature?		Yes/No/NA
Would you be willing to save your vaccination in the app?		Yes/No/NA
Would you share it with health authorities?		Yes/No/NA
In the following, you can share your reasons for not wanting to share your vaccination with health authorities through the app.		

Question group: Personal and closing questions	
How old are you?	18-24, 25-34, 35-44, 45-54, 55-64, 65-74 years old, older than 74.
Do you own a smartphone?	Yes/No/NA
Do you use the current Corona-Warn-App?	Yes/No/NA
Do you find the current Corona-Warn-App useful?	1-7
Please answer the following questions regarding the advanced Corona-Warn-App presented here.<br>… the extension of the app is useful.	1-7
… the service provider of this App is trustworthy.	1-7
… sharing my data with the Corona-Warn-App involves a high privacy risk for me.	1-7
… in the future, I would use the Corona-Warn-App if it includes the features presented here.	1-7
… I would record other vital parameters (pulse. stress level. etc.) in the Corona-Warn-App if this would create additional functionalities.	1-7
What sources of information do you use to stay up to date on the pandemic?	Family<br>Friends<br>Job<br>TV News<br>Radio News<br>Newspaper<br>Social Media<br>Podcast<br>Youtube
Do you have further suggestions for improving the prototype?	
